# Editorial: Microalgae-microbe interactions: advances and applications

**DOI:** 10.3389/fmicb.2026.1815619

**Published:** 2026-03-18

**Authors:** Ankita Srivastava, Minsik Kim, Sang-Ah Lee, Jin-Ho Yun

**Affiliations:** 1 Department of Botany, Faculty of Science, Siddharth University, Kapilvastu, India; 2 Department of Biotechnology, Inha University, Incheon, Republic of Korea; 3 Faculty of Biotechnology, College of Applied Life Sciences, Jeju National University, Jeju, Republic of Korea; 4 Bio-Health Materials Core-Facility Center, Jeju National University, Jeju, Republic of Korea; 5 Interdisciplinary Graduate Program in Advance Convergence Technology and Science, Jeju National University, Jeju, Republic of Korea; 6 Cell Factory Research Center, Korea Research Institute of Bioscience and Biotechnology, Daejeon, Republic of Korea; 7 Department of Bioresource and Environmental Engineering, School of Biotechnology, Korea Research Institute of Bioscience and Biotechnology, University of Science and Technology, Daejeon, Republic of Korea; 8 Department of Integrative Biotechnology, Sungkyunkwan University, Suwon, Gyeonggi-do, Republic of Korea; 9 Bigelow Laboratory for Ocean Sciences, East Boothbay, ME, United States

**Keywords:** bacterial consortia, microalgae-microbe interactions, microbial ecology, phycosphere dynamics, synthetic biology

Microalgal interactions within microbial communities across different environmental gradients and physical scales are widely acknowledged to play essential roles in microbial evolution and ecosystem functions, and are further recognized as key drivers of productivity, resilience, and functionality in microalgae-based biotechnological systems. These interactions—spanning molecular to population levels—influence the production and transport of key molecular cargoes with broader ecological and engineering implications, and biogeochemical cycling on a global scale.

Recent advancements in multi-omics approaches and synthetic biology have significantly expanded our understanding of metabolic versatility and the interactive mechanisms underlying diverse microalgae-microbe consortia. Nevertheless, the wide spectrum of possible interactions in these consortia urgently calls for further systematic investigations to unravel novel biological insights and create opportunities for advancing microalgal synthetic biology, nature-based solutions for bioremediation and carbon sequestration, and the commercialization of biofuels and high-value bioproducts. Acknowledging the vital importance of microalgae-microbe interactions, this Research Topic was established to present a collection of recent advances in the field. Seven articles in this Research Topic cover a broad range of topics—from reports on novel microbes influencing bloom-forming algae and elusive predatory bacteria in outdoor microalgal cultivation systems to a deep learning-based predictive model for chloroplast-targeting peptides and a timely review on microbiome engineering approaches for algal biotechnology.

Mankiewicz-Boczek et al. investigated the bacterial consortia associated with morphologically distinct cyanobacteria that can influence the ecological threat of harmful algal blooms across freshwater bodies globally. The results of 16S rRNA amplicon sequencing and shotgun metagenomics supported that phycosphere composition and nutrient-cycling capacity depended on the dominant cyanobacterial strain, with *Microcystis* supporting more complex bacterial associations than *Aphanizomenon* and *Planktothrix*. Interestingly, non-diazotrophic blooms exhibited greater abundances of nitrogen- and phosphorus-cycling genes, indicating their importance in bloom's ecological impact possibly via nutrient transformations. Resonating with these findings, Li et al. reported bacterial strains isolated from blooms of *Phaeocystis globosa* in marine ecosystems and highlighted the role of ammonifying and phosphorus-solubilizing functions in supporting the growth of this bloom-forming alga. A particular isolate, GXAS306^T^, was identified as belonging to the genus *Aliikangiella*, and its growth-promoting activity reiterates that the fate of bloom-forming algae is closely influenced by associated bacterial communities.

While bloom-forming algae also include macroalgae, such as seaweeds that form large-scale green tides, Liu et al. investigated the impacts of decaying *Ulva* green tides on the phycospheric microbiome. Shotgun metagenomic analysis suggested that the demise of *Ulva prolifera* green tides substantially altered phycospheric microbial community structure. Specifically, the microbial community was dominated by Proteobacteria and Bacteroidota and exhibited increased abundances of algae-lysing bacteria. These taxonomic shifts were accompanied by a decrease in microbial diversity, along with an increased abundance of carbohydrate-active enzymes involved in polysaccharide degradation and the enrichment of nitrogen metabolism genes. In addition to *in situ* microbes, microbes obtained from non-aquatic environments can also exert strong influences on microalgae. As a case in point, Du et al. reported algicidal activity of *Streptomyces* sp. LMJ-114, isolated from soil samples, against both *Microcystis aeruginosa* and filamentous cyanobacteria. 16S rRNA sequencing indicated the highest similarity of this novel isolate to *Streptomyces jiujangensis* JXJ 0074T, and algicidal activity against *M. aeruginosa* was associated with elevated ROS production and lipid peroxidation, along with reduced production of microcystin. Subsequent NMR analyses identified L-valine as a major algicidal compound, and the findings may contribute to the development of environment-friendly bloom mitigation strategies.

Moving beyond natural systems, Nguyen et al. re-established a microalgae-bacteria pest model through continuous passage in the laboratory. The authors obtained a previously characterized FD111-like pest from open ponds of *Nannochloropsis* and passaged two infection sources into cultures of *Nannochloropsis oceanica*. Passage into healthy cultures resulted in infection throughout 10 passage cycles, with microalgal deterioration occurring after 4 days in each cycle. Molecular assays determined that this FD111-like bacterium belongs to the order *Oligoflexales*, possibly the genus *Pseudobacteriovorax*; further TEM imaging indicated that physical attachment of the FD111-like bacterium initiates algal predation. Notably, even though this predatory bacterium was not dominant within the broader bacterial community throughout continual passages, its low abundance was sufficient to crash microalgal cultures, underscoring the need for effective crop protection strategies to address elusive bacterial predators in industrial algal cultivation systems.

In recognition of the industrial relevance of microalgae-microbe interactions, Koneru et al. critically reviewed the ecological and biotechnological potential of algal microbiome. With advances in synthetic biology and ecological approaches, algal microbiome engineering has become increasingly viable, offering opportunities to harness beneficial algal-microbiome interactions. The authors emphasized the potential of synthetic biology and machine learning to enable precise management of large-scale algal cultivation, as well as to support sustainable solutions to global challenges through microbiomes tailored to specific industrial objectives. Nonetheless, incomplete understanding of the molecular-to-ecological mechanisms underlying algal-microbiome interactions, coupled with the species-specificity of these interactions, highlights the need for general theoretical frameworks as well as mature tools and techniques for microbiome engineering. As a relevant enabling case study, Choi et al. reported an improved deep learning model for binary classification of chloroplast transit peptides (cTPs) and non-cTPs, trained on datasets compiled from the literature on *Chlamydomonas reinhardtii*. Many chloroplast proteins are nuclear encoded and imported into chloroplasts after translation; therefore, predicting cTPs is critical for chloroplast-targeted engineering as well as for elucidating evolutionary histories. Dubbed Chlamy_ChloroPred, the proposed classifier achieved substantially higher prediction accuracy than the previously reported TargetP 2.0. Nevertheless, the remaining room for improvement in model performance aligns with Koneru et al., underscoring the need for larger, higher-quality biological datasets to further advance predictive models and better understand—and ultimately exploit—microalgae-microbe interactions.

Although no single Research Topic can cover the full breadth of our topic, the seven articles disseminated herein represent its transdisciplinary nature. With growing excitement around new tools and approaches, we remain optimistic that our collective understanding of the intricate tapestry of microalgae-microbe interactions will only deepen, as the articles in this Research Topic demonstrate. With seemingly intractable yet intriguing challenges remaining, we strongly encourage researchers with fresh perspectives and ideas to further explore this exciting area.

